# Beyond VOR gain: the functional head impulse test as a probe of multisensory integration and gaze stabilization

**DOI:** 10.3389/fneur.2026.1913947

**Published:** 2026-09-03

**Authors:** Stefano Ramat, Bruna Maria Vittoria Guerra, Marco Mandalà

**Affiliations:** 1 Department of Computer, Industrial and Biomedical Engineering, University of Pavia, Pavia, Italy; 2 Department of Medicine, Surgery and Neuroscience, Neurology and Clinical Neurophysiology Section, University of Siena, Siena, Italy

**Keywords:** dynamic visual acuity, fall risk, functional head impulse test, gaze stabilization, multisensory integration, vestibular migraine, vestibular testing, vestibulo-ocular reflex

## Abstract

The functional head impulse test (fHIT) measures what individuals can see during high-acceleration passive head impulses, quantifying the percentage of correctly identified briefly flashed optotypes rather than inferring gaze stabilization from kinematic eye velocity. This paper traces the technological evolution of fHIT from its conceptual origins in dynamic visual acuity testing to its standardized protocol (80-ms presentation at peak head acceleration binned across 1,000–7,000°/s^2^) and leverages clinical data to propose a new conceptual framework for functional vestibular assessment. Accumulated evidence reveals a stark dissociation between video head impulse test (vHIT) kinematic gain and fHIT functional performance, proving that the clinical bottleneck in real-world vestibular impairment is frequently the central capacity to integrate multisensory signals under visual and cognitive demands in real time. In peripheral vestibulopathies, fHIT demonstrates that functional vision depends entirely on the precise temporal clustering of covert saccades within the 80-ms optotype presentation window. In vestibular migraine, fHIT under optokinetic conflict could unmask central sensory re-weighting failures (sensory mismatch) completely missed by kinematic testing. In older people, fHIT performance—unlike vHIT gain—correlates with Tinetti Balance Scores, predicting fall risk with an odds ratio of 3.9, while dual-task studies confirm that concurrent cognitive load selectively degrades fHIT accuracy. Based on these converging dissociations, we propose a novel three-level functional framework that organizes gaze stabilization into peripheral reflex mechanics, saccadic substitution, and central sensory gating. This model establishes that the fHIT could directly probe the higher-level central integrative loop by simultaneously challenging gaze stabilization, visual discrimination, attentional allocation, and short-term memory encoding during unpredictable passive perturbations. This theoretical shift reframes abnormal functional performance alongside normal kinematic gain not as a paradox, but as an indicator of central processing or cognitive resource allocation/attention deficits, providing a new blueprint for tailoring advanced vestibular rehabilitation.

## 1 Introduction

The vestibulo-ocular reflex (VOR) represents the primary neural mechanism for stabilizing retinal images during rapid head rotations, generating compensatory eye movements in the direction opposite to head motion. Operating best at frequencies above 0.05 Hz and up to 5 Hz or more, where visual tracking and cervico-ocular contributions are inadequate, the VOR is essential for maintaining visual acuity during natural activities such as walking, running, and rapid head turns. High-frequency VOR function, characterized by head accelerations exceeding 3,000°/s^2^, is critical for daily functional performance, yet traditional vestibular assessment methods, including caloric testing and rotary chair testing, evaluate only low-frequency VOR function (0.003–0.5 Hz) and may fail to detect deficits in the physiologically relevant high-frequency range (1).

The clinical head impulse test (HIT), introduced by Halmagyi and Curthoys in 1988, revolutionized bedside vestibular assessment by exploiting the push-pull principle of semicircular canal function and detecting catch-up saccades following brief, unpredictable, high-acceleration head rotations (overt catch-up saccades) (2). The subsequent development of video head impulse testing (vHIT) in the early 2000s enabled the spread of quantitative measurement of VOR gain, i.e. the ratio of eye velocity to head velocity, beyond the few laboratories equipped with a search coil apparatus. The vHIT also allowed the identification of both overt (occurring after head rotation) and covert saccades (occurring during head rotation), the latter being almost imperceptible to clinical observation and potentially masking peripheral vestibular deficits (3, 4).

Nonetheless, vHIT is designed to perform a purely kinematic measure: it quantifies how well eye velocity compensates for head velocity but does not directly assess whether the residual VOR is sufficient to preserve functional vision during natural head movements. As even a few degrees per second of retinal slip seriously impairs visual acuity, the functional effectiveness of gaze stabilization, what the patient can actually see during head motion, may differ substantially from the measured gain (5). Compensatory mechanisms including predictive saccades, covert saccades, and cervico-ocular reflexes may preserve gaze stabilization despite reduced VOR gain, creating a dissociation between kinematic performance and functional visual capability (6).

The functional head impulse test (fHIT) was developed to address this fundamental limitation by directly measuring dynamic visual acuity, the ability to recognize visual targets during head impulses, rather than inferring functional performance from kinematic measurements such as the gain of the reflex (7). This review traces the technological and psychophysical evolution of functional vestibular testing, mapping the trajectory from early dynamic visual acuity paradigms through the head impulse testing device (HITD) to standardized fHIT protocols. Synthesizing clinical evidence from 2018–2026 across diverse patient cohorts, we propose a novel framework that reframes the fHIT as a comprehensive probe of real-time functional gaze stabilization, multimodal integration, and cognitive resource allocation.

## 2 Historical development of functional VOR testing

The VOR operates through a three-neuron arc connecting semicircular canal afferents to extraocular motor neurons, generating compensatory eye movements with latencies of as low as 5–7 milliseconds. This rapid response is essential for gaze stabilization during high-frequency, high-acceleration head movements that characterize natural human motion. The clinical HIT, by delivering brief (100–200 ms), unpredictable, high-acceleration (3,000–6,000°/s^2^) head rotations in the plane of individual semicircular canals, provides a physiologically relevant stimulus that cannot be anticipated or compensated by predictive mechanisms.

Video head impulse testing advanced clinical assessment by enabling quantitative measurement of VOR gain and precise identification of compensatory saccades. High-speed cameras (typically 220–250 Hz) track eye position during head impulses, allowing calculation of instantaneous eye and head velocities and identification of saccades with millisecond precision. The detection of covert saccades, compensatory eye movements occurring during rather than after head rotation, represented a significant advance, as the presence of these saccades, often clinically imperceptible, are an indicator of reduced VOR gain.

To bridge the gap between kinematic measurement and patient reports of their symptoms, dynamic visual acuity (DVA) tests emerged in the late 1980s and gained widespread adoption in the early 2000s as patient-centered assessments of VOR efficacy. The foundational work of Longridge and Mallinson in 1987 introduced the “dynamic illegible E-test,” which compared static visual acuity with acuity during passive head oscillations, establishing the principle that vestibular deficits manifest as a loss of visual discrimination during head motion (8).

Herdman and colleagues subsequently developed computerized DVA systems in which subjects identified the orientation of an “E” optotype displayed during active, sinusoidal head rotations at 1–2 Hz when head velocity reached 120–180°/s, with the size of the optotype being progressively decreased until recognition failed. A decrement of ≥0.3 logMAR relative to static acuity was considered pathological. These early DVA paradigms generally demonstrated clinical reliability in detecting unilateral and bilateral vestibular hypofunction and provided a functional measure that correlated with patient-reported oscillopsia (9).

However, active head movements inherent in these protocols exposed a fundamental limitation: the central nervous system can exploit efference copy signals from neck motor commands to activate predictive gaze-stabilization mechanisms that do not rely on peripheral vestibular input, thereby potentially masking residual canal deficits in well-compensated people. Della Santina and colleagues demonstrated in 2002 that apparent VOR gain during active head movements was significantly higher than during passive, unpredictable stimuli in people with unilateral labyrinthectomy, confirming that voluntary motion allows central preprogramming to supplement or replace vestibular-driven eye movements (10).

Recognition of these confounds motivated the development of passive DVA paradigms. Schubert and colleagues introduced a version of DVA using passive head thrusts in canal planes, presenting the “E” optotype when head velocity exceeded 120–180°/s for at least 40 ms, and demonstrated that this approach yielded VOR-dependent performance that inversely correlated with quantitative VOR gain measured by scleral search coils in people with superior canal dehiscence and unilateral vestibular deafferentation (11). Nonetheless, even passive DVA approaches remained limited by relatively low head accelerations, prolonged testing duration (often requiring ~100 impulses), and reliance on velocity thresholds that do not capture the full physiological range of high-frequency canal function.

In parallel, Goebel and colleagues introduced the Gaze Stabilization Test (GST) in 2007, which adaptively determined the peak head velocity at which subjects could discern a fixed-size optotype (0.3 logMAR above static acuity) during active head shaking, demonstrating 93% specificity and 64% sensitivity for unilateral vestibular dysfunction (12). However, the use of active, predictable head movements again limited its diagnostic power in compensated people, and surprisingly, normative data for GST remained sparse.

A critical methodological advance came from Vital and colleagues in 2010, who developed a DVA test using Landolt rings with eight possible orientations (reducing chance performance from 25% to 12.5% relative to the four-orientation “E” optotype) and implementing a QUEST adaptive algorithm that converged on the visual acuity threshold in as few as 20 trials (13). This paradigm used passive head impulses with velocity thresholds >150°/s and demonstrated sensitivity and specificity comparable to search-coil HIT measurements, while substantially reducing testing time and burden. These refinements set the stage for the transition from DVA as a generic functional measure to a test specifically optimized for high-acceleration, canal-specific VOR assessment.

The head impulse testing device (HITD), introduced by Ramat, Mandalà, Versino, Colnaghi, and colleagues in 2010–2012, represented a conceptual shift from velocity-threshold DVA to acceleration-based functional testing (7, 14). Recognizing that compensatory mechanisms and covert saccades may preserve kinematic VOR gain while leaving people functionally impaired, the HITD was designed to assess whether the VOR could maintain clear vision across a spectrum of head angular accelerations ranging from 2,000 to 7,000°/s^2^, independently of each subject's baseline visual acuity.

The system employed a lightweight inertial measurement unit (three-axis accelerometer and single-axis gyroscope) mounted on the subject's head, acquiring data at 10 kHz and presenting Sloan letters for approximately 33 ms when head angular acceleration exceeded a user-defined threshold. Crucially, the optotype size was fixed at 0.8 logMAR above each individual's static visual acuity, ensuring that all subjects faced an equally challenging visual task regardless of their baseline vision, and the outcome was simply the percentage of correct identifications at each acceleration bin.

Initial validation in 22 healthy controls showed near-perfect performance (~98% correct answers) across all accelerations, while 30 of 39 participants with various vestibular disorders (vestibular neuritis, Ménière disease, unilateral and bilateral vestibulopathy, central vestibular disorders) showed abnormal HITD results, with abnormalities most pronounced at higher accelerations (>4,000°/s^2^). Notably, HITD detected significantly more abnormalities than clinical HIT in participants with central vestibular deficits, likely because covert saccades, though kinematically effective, induce functional blindness (saccadic suppression) that prevents optotype recognition in a test configuration in which the letter was displayed for a very short time interval (7). This observation underscored the fundamental distinction between compensatory eye movements and functional vision.

Building on the HITD framework, Colagiorgio and colleagues in 2013 implemented the HITD as a paradigm on the high-speed video-oculography EyeSeeCam system, and added a photodiode applied to the screen for the verification of stimulus timing. This was intended as a research tool enabling direct comparison of VOR gain, saccade patterns, gaze stability (computed as head position plus eye position), and optotype recognition (15). This “new tool for investigating the functional testing of the VOR” allowed researchers to dissect the relationship between covert saccades, retinal slip velocity, and reading errors, revealing that successful optotype identification required gaze stability within a narrow window during the 80-ms presentation interval. By measuring the true timing performance of the experimental setup and analyzing individual trials, this approach elucidated which parameters, i.e. stimulus duration, delay relative to peak acceleration, and optotype size increment above static acuity, were critical for optimizing diagnostic sensitivity. The same research system was used by Ramaioli and colleagues to investigate the effect of an opioid (Remifentanil) on VOR function in a group of 14 healthy men and comparing the induced changes in term of both VOR gain and pca (16). They found that a patient with VOR gain of 0.6, yet systematically producing covert saccades, was able to effectively stabilize gaze during the critical period of visual processing and had therefore excellent functional vision, while another patient with identical gain but generating delayed saccades experienced severe oscillopsia. In this study the first evidence for a functional effectiveness of covert saccades was found in the above-mentioned patient having low VOR gain at vHIT yet high pca with HITD testing.

The evolution from HITD to the functional head impulse test (fHIT) involved both hardware refinement and standardization of psychophysical protocols. Corallo, Versino, Mandalà, and colleagues reported preliminary fHIT data in 2018, using Landolt C rings (eight orientations) displayed for 80 ms at peak head acceleration during passive, unpredictable impulses in the planes of the horizontal, left-anterior–right-posterior (LARP), and right-anterior–left-posterior (RALP) semicircular canals (17). Because the target vanishes after 80 ms, any compensatory or catch-up saccades generated by the central nervous system must be timed with millisecond precision. If a saccade reduces gaze position error but lands after the 80-ms offset, it contributes nothing to functional vision; conversely, short-latency covert saccades that cluster directly within this gray window can rescue target recognition even in the presence of a severely degraded peripheral VOR drive. This temporal bottleneck underpins why fHIT tracks real-world visual performance far more closely than standard kinematic measures.

In healthy young adults, normative %CA values ranged from approximately 85% to >90% in the lateral canals and slightly higher in the vertical canals, with no significant difference between sides but modest sex-related differences at the highest accelerations (18). Subsequent studies validated fHIT's ability to distinguish acute from compensated vestibular neuritis, detect bilateral vestibulopathy, and quantify oscillopsia severity. Critically, fHIT performance moderately correlated with patient-reported oscillopsia questionnaires, whereas treadmill-based DVA did not, and all people could complete fHIT while nearly half could not complete treadmill DVA at all speeds (19).

The standardized fHIT protocol involves several key methodological elements:

- 1. Static visual acuity baseline: First measured using a QUEST adaptive algorithm to establish each individual's baseline visual capability. Critically, optotypes are presented for 80 ms during static testing, the same duration used for dynamic testing, ensuring that any performance differences reflect VOR function rather than differential temporal processing demands.
- 2. Dynamic optotype sizing: Set at a fixed increment (typically 0.6 logMAR) above the static threshold to ensure equivalent task difficulty across subjects.
- 3. Optotype characteristics: Landolt C rings with eight possible orientations.
- 4. Presentation timing: 80-ms display duration at peak head acceleration.
- 5. Acceleration range: Testing across bins spanning 1,000–7,000°/s^2^, with diagnostic focus on 4,000–6,000°/s^2^.
- 6. Canal-specific testing: Separate assessment of horizontal, LARP, and RALP canal planes.
- 7. Outcome measure: Percentage of correct answers (%CA) for leftward and rightward (or upward and downward) head impulses.

## 3 Clinical applications of fHIT

### 3.1 Peripheral vestibular disorders

fHIT and vHIT are complementary rather than redundant in peripheral vestibular disease. In vestibular neuritis, both tests correctly lateralize the lesion acutely and track compensation over 3 months, but fHIT consistently shows larger asymmetry indices than vHIT and detects more residual abnormalities at follow-up, even after vHIT gain has partially normalized through central compensation (17). Versino and colleagues directly compared the two techniques in vestibular neuritis individuals at onset and at 3 months, finding that vHIT and fHIT data did not correlate with each other, and that fHIT detected more abnormalities than vHIT with lower %CA values than VOR gain at both timepoints (20). Asymmetry indices were significantly larger for fHIT than for vHIT, with significant effects for side (affected side lower than healthy), time (onset lower than 3 months), and technology (fHIT lower than vHIT). Notably, neither test correlated significantly with Dizziness Handicap Inventory scores, suggesting that functional gaze stabilization deficits captured by fHIT represent a distinct dimension of vestibular impairment from the patient's subjective disability experience. The pattern of divergence between vHIT and fHIT is itself diagnostically informative: when vHIT gain improves faster than fHIT %CA, it signals that kinematic compensation via covert saccades is occurring but that the timing of those saccades is not yet sufficient to preserve vision within the 80 ms optotype window. The critical variable is not the presence of covert saccades but their latency relative to stimulus onset.

Casani et al. (21) extended this to participants evaluated retrospectively after acute symptom resolution, comparing 20 subjects with acute unilateral vestibulopathy (AUV), 25 vestibular migraine subjects, and 20 healthy controls. In AUV people, fHIT revealed significant %CA differences between the deficit and normal sides both without and with a competing optokinetic screen (*p* < 0.001 for both conditions). The mean %CA difference with versus without the confounding stimulus (ΔCS) was 10.4 in AUV subjects versus 2.15 in controls (*p* < 0.001), demonstrating that even after acute symptoms resolve, functional VOR deficits persist and are unmasked by visual conflict. This result argues for using fHIT with optokinetic challenge as a standard follow-up tool after acute unilateral vestibulopathy, particularly when kinematic testing appears normal or near-normal (21).

In bilateral vestibulopathy, where sophisticated compensatory strategies may partially obscure kinematic deficits, van Dooren et al. (19) demonstrated that fHIT performance correlated moderately with patient-reported oscillopsia severity (measured by the Oscillopsia Severity Questionnaire, *r* = −0.56 for rightward rotations), while kinematic gain did not. Interestingly, among 23 subjects 78% showed an abnormal fHIT to both sides, 4 participants (17%) had normal fHIT outcomes and 1 had a unilateral abnormal fHIT.

All bilateral vestibulopathy participants in that study completed fHIT testing, whereas nearly half could not perform treadmill-based dynamic visual acuity at all speeds due to balance risk and fall hazard. People with similar vHIT gains showed vastly different oscillopsia levels depending on the effectiveness and timing of their compensatory saccades, a dissociation fHIT captures and treadmill DVA does not.

### 3.2 The active vs. passive impulse distinction

**Figure 1:**
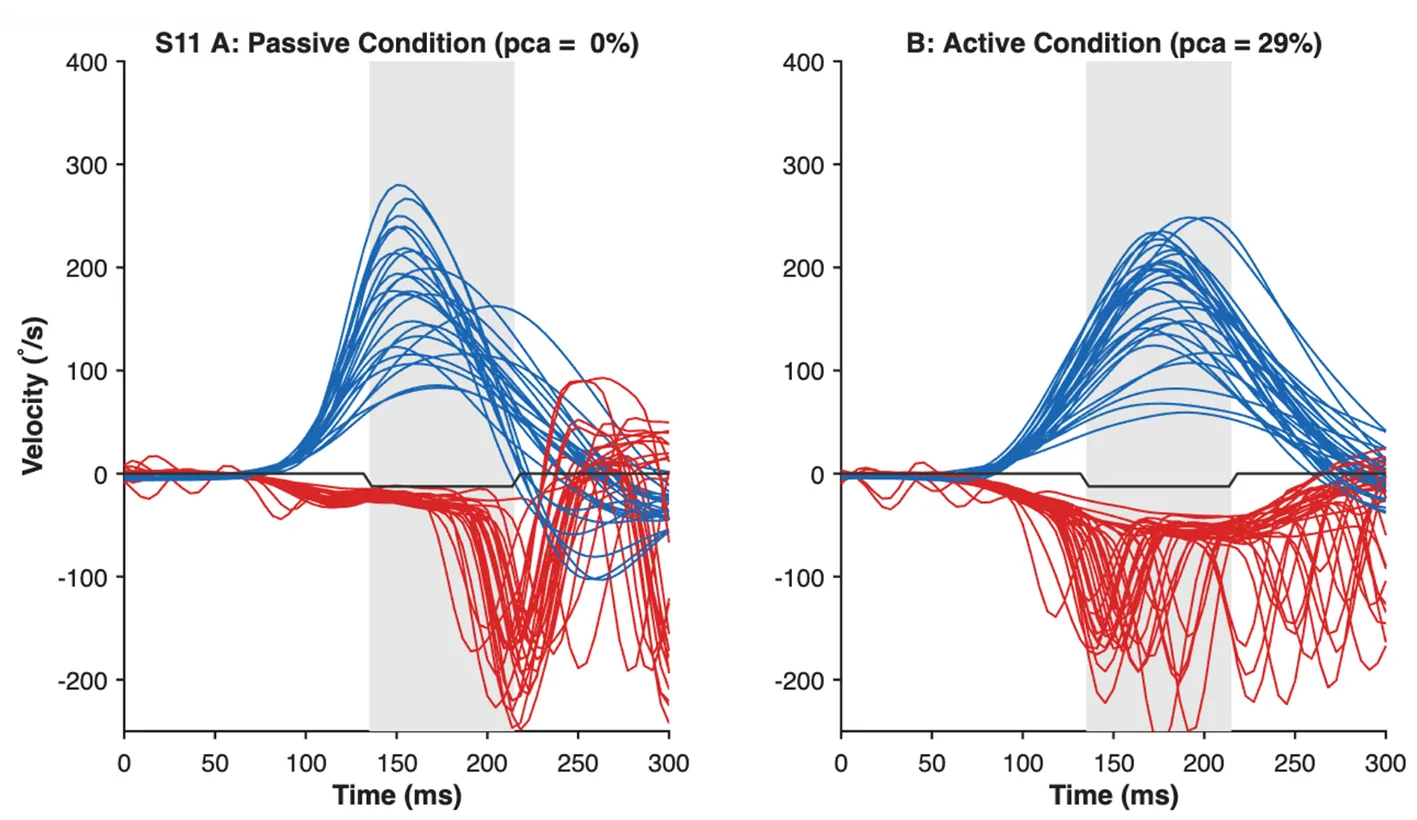
Raw eye-head kinematic traces and functional performance profiles during testing of the Vestibulo-Ocular Reflex (VOR) in a single subject [Unpublished data from (15)]. The vertical gray shaded region across both panels explicitly defines the 80-ms perceptual window during which the Landolt C optotype is visible on screen. **(A)** Passive Condition: Traces obtained during examiner-delivered, unpredictable head impulses. Eye velocity (red) poorly matches head velocity (blue) during the critical 80-ms optotype presentation window, resulting in a compromised percentage of correct answers (pca). **(B)** Active Condition: Traces obtained during self-generated head impulses. Gaze-stabilization is improvedduring the visual window, leading to a substantial increase of correct answers. This recovery is driven by central pre-programming and an efference copy command, which triggers ultra-short-latency covert catch-up saccades that arrive precisely inside the 80-ms viewing block to minimize retinal slip.

Sjögren et al. (6) used the HITD-FT device to provide a mechanistic insight with direct clinical relevance by comparing active (self-generated) and passive (examiner-delivered) head impulses in eight subjects with complete unilateral vestibular loss (four men, four women; mean age 47 years). Active impulses to the lesioned side produced fHIT performance equivalent to that on the intact side (*p* = 0.016). Detailed analysis revealed that active impulses resulted in significantly smaller gaze position errors during the visual perception window compared to passive impulses (*p* = 0.006), and that this position error reduction correlated strongly with shorter latencies of the first saccade (*p* < 0.001). The mechanism is pre-programming of short-latency covert saccades via efference copy signals from the motor command generating the head movement: these saccades arrive during rather than after the 80 ms perceptual window, stabilizing gaze sufficiently for optotype recognition despite the absent VOR drive. Passive impulses eliminate this efference copy advantage and expose the true functional deficit. This independent finding by the Lund University group explains why subjects with unilateral vestibular loss frequently report better functional vision during self-generated daily movements than during unexpected perturbations, and it provides scientific justification for fHIT's use of passive unpredictable impulses: to measure VOR-dependent gaze stabilization without the confound of anticipatory motor programming (Figure 1).

### 3.3 Multisensory integration: vestibular migraine and visual conflict

**Figure 2:**
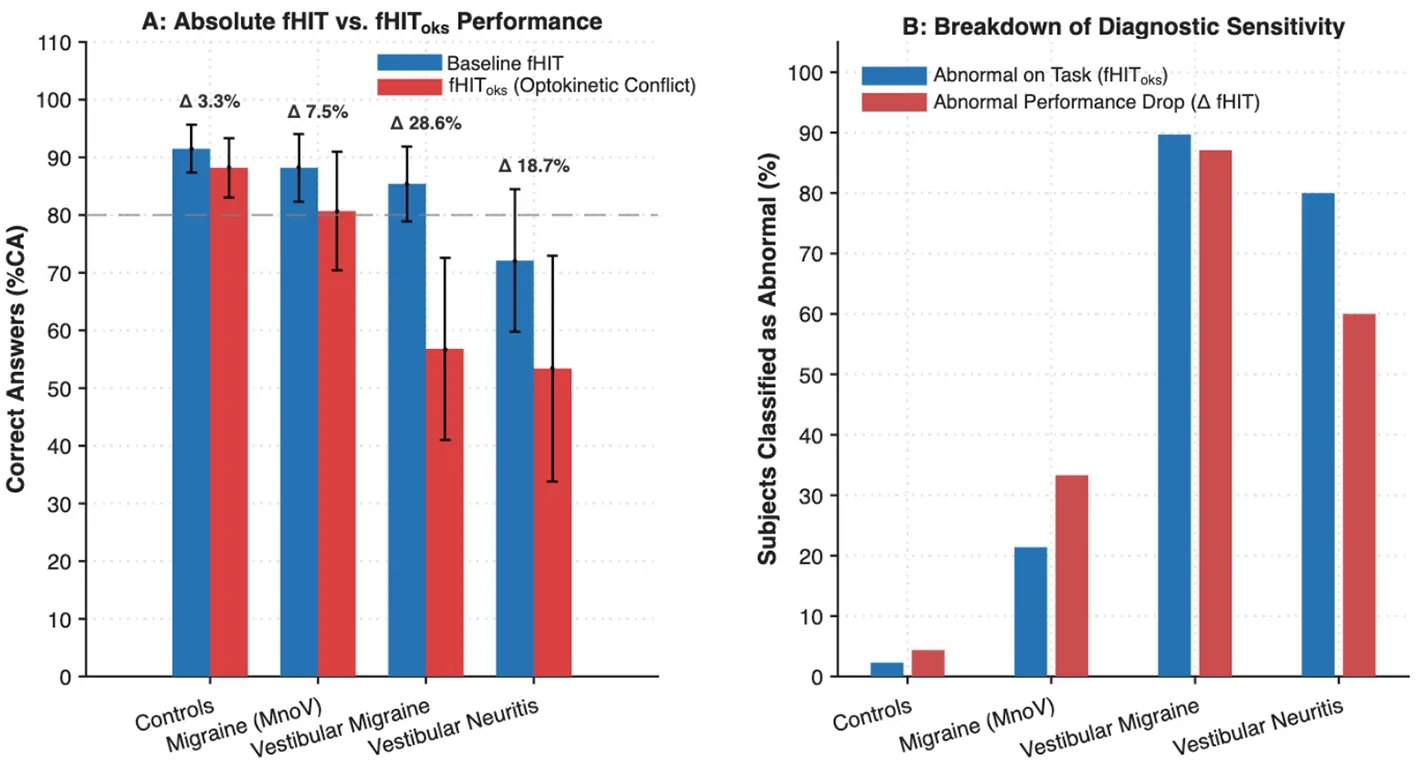
Quantifying central multisensory processing deficits under visuo-vestibular conflict across clinical cohorts. Data adapted from Versino, Mandalà et al. (2020). **(A)** Performance cost of full-field visual noise, showing the results from standard testing and with optokinetic stimulation and the mean difference in the percentage of correct answers (Δ Correct Answers = fHIT - fHIT_oks_). Controls (Ctrl) and non-vestibular migraineurs (MnoV) exhibit minimal degradation, whereas Vestibular Migraine (VM) subjects manifest a severe performance drop. **(B)** Diagnostic sensitivity and clinical abnormality rates based on normal cut-offs (mean ± 2.5 SD from controls). The blue bars track subjects classified as abnormal on the fHIT_oks_ task itself; the red bars represent participants with an abnormally large drop-off (ΔfHIT). While Vestibular Neuritis (VN) subjects score poorly due to a structural peripheral arc deficit, the selective spike in the red series for the VM group isolates a distinctive failure of central sensory re-weighting and visual conflict gating.

To try to isolate central multisensory integration and sensory re-weighting capabilities, the standardized fHIT framework can be modified to introduce a visuo-vestibular mismatch paradigm via full-field optokinetic stimulation (fHIT_oks_) (22). In this configuration, while the patient undergoes the standard passive, high-acceleration head impulse in the yaw plane, the monitor simultaneously presents a dense background cloud of pseudo-randomly distributed, high-contrast dots (23). This visual background rotates uniformly in the frontal plane around the central optotype at a constant angular velocity of 36°/s. This setup generates an acute sensory conflict where the angular vestibular drive (yaw plane) must be prioritized to facilitate retinal stabilization, while the disruptive, large-field visual motion drive (roll plane) must be centrally suppressed or de-weighted to prevent degradation of visual acuity during the 80-ms optotype display window. The fHIT optokinetic conflict paradigm introduced by Versino and colleagues in 2020 revealed a qualitatively different profile of failure in vestibular migraine compared to peripheral vestibular disorders, one that cannot be explained by reduced VOR gain (Figure 2). In that study, 39 vestibular migraine (VM) subjects, 42 migraine-without-vertigo subjects (MnoV), 15 vestibular neuritis (VN) subjects, and 44 controls performed standard fHIT and fHIT under full-field frontal-plane optokinetic stimulation. While VN subjects failed mainly due to reduced VOR gain, VM participants failed specifically under visual conflict: 87% of VM subjects showed an abnormally large %CA drop under optokinetic stimulation, compared to approximately 60% in VN, 33% in MnoV, and a modest decrement in controls (23). Casani et al., in 2021, reported similar outcomes among 25 VM subjects: a mean difference in the errors without and with the optokinetic background of 18.36%. Around 60% of VM individuals showed a significant decrease, intended as greater than 2 standard deviations of the means, in their %CA with the optokinetic stimulation (21). Since VOR gain is typically preserved in VM, this pattern localizes the impairment to the central mechanism that weights and reconciles conflicting vestibular and visual motion signals, a known domain of migraine pathophysiology involving cortical hyperexcitability and abnormal multisensory gain control. The MnoV intermediate profile suggests a continuum of multisensory integration impairment across the migraine spectrum rather than a discrete category boundary.

This is corroborated by the work of Konukseven and colleagues, who demonstrated reduced fHIT performance in migraine participants without any overt vestibular symptoms compared to healthy controls, suggesting that vestibular processing abnormalities may be present sub-clinically across the migraine population and may contribute to symptoms such as motion sensitivity, photophobia, and phonophobia even in the absence of frank vertigo (24). The fHIT with optokinetic conflict currently represents the only available test that dissociates peripheral VOR failure from central multisensory re-weighting failure based on a qualitative pattern, the profile of performance degradation under conflict.

The fHIT paradigm with optokinetic stimuli has been indicated by an international panel of VM experts as a promising test for the diagnosis of VM (25).

### 3.4 Aging, falls, and cognitive load

Age-related decline in fHIT %CA progresses continuously across adulthood, with a study documenting a significant negative correlation between age and %CA (*r* = −0.311) in healthy individuals with no diagnosed vestibular pathology (26). This decline reflects converging contributions of peripheral vestibular senescence (progressive hair cell and primary afferent loss), central VOR processing changes, reduced saccadic velocity and accuracy, and declining multisensory integration capacity. Importantly, a %CA value that would be clearly pathological in a young adult may fall within the age-appropriate normal range in an older individual, making age-matched normative reference values essential for clinical interpretation. Conversely, an older individual with normal vHIT gain may have functionally significant VOR deficits detectable only by fHIT.

The clinical consequence of this age-related decline was quantified by Politi et al. (27) in a study comparing 31 participants over 65 years with a history of falls to 19 age-matched controls without falls. Mean fHIT %CA was significantly lower in fallers (66.40% ± 5.12) than in controls (76.79% ± 7.04; *p* = 0.029). In contrast, vHIT VOR gain showed no significant difference between groups (0.97 ± 0.04 in cases versus 0.87 ± 0.05 in controls; *p* = 0.131). Critically, a linear correlation was found between fHIT performance and the Tinetti Balance Score, while no such correlation existed for vHIT gain. Subjects with %CA below 80% had an odds ratio of 3.9 for having experienced a fall in the previous year (27). This dissociation is not simply a matter of test sensitivity: it reflects the nature of falls in older people, which occur during complex, multisensory, divided-attention situations rather than during isolated high-frequency VOR demands. fHIT, by requiring visual discrimination under passive unpredictable head motion, reproduces a miniaturized version of these real-world integration demands in a way that pure kinematic testing cannot.

This interpretation is reinforced by the dual-task findings of Sezer & Baydan Aran, who showed that adding a concurrent cognitive task, verbal fluency in semantic and phonemic categories, significantly reduced fHIT %CA across 27 healthy adults, while leaving vHIT gain statistically unchanged (28). The cognitive load imposed by concurrent verbal processing competed for the attentional resources and working memory required to perceive, hold, and report the briefly flashed Landolt C orientation. Together, these findings indicate that fHIT performance in older people reflects not only peripheral vestibular status but the central integrative capacity to allocate attention and process visual information under competing demands, precisely the capacity indexed by the Tinetti Balance Score and predictive of fall risk. Clinicians interpreting fHIT in older people or cognitively impaired subjects should recognize that reduced %CA may reflect a central integration bottleneck rather than, or in addition to, peripheral vestibular loss.

### 3.5 Neurodevelopmental and neurological conditions

fHIT has identified vestibular dysfunction in populations where conventional testing is difficult or where involvement would not be predicted by the primary diagnosis. A 2020 study found significantly reduced %CA with the fHIT in children with neurodevelopmental disorders (autism spectrum disorder, ADHD and reading impairment) compared to neurotypical controls (*p* < 0.0001), while no significant differences were found among the three clinical subgroups of neurodevelopmental disorders, suggesting a shared vestibular processing vulnerability rather than condition-specific profiles (29). This finding has implications for understanding the neurobiological basis of these conditions: vestibular input contributes critically to spatial cognition, motor planning, and multisensory integration, all of which are affected in neurodevelopmental disorders. The brief, passive, game-like nature of fHIT offers a practical testing advantage in this population, as traditional vestibular assessments requiring sustained cooperation, caloric irrigation, prolonged rotary chair protocols, are poorly tolerated by children with neurodevelopmental disorders.

The Turkish Neuro-Otology group of Karababa and colleagues demonstrated significant reduction of fHIT performance in Parkinson's disease patients compared to age-matched controls: the mean %CA in the lateral SCC plane was below 60% (30). Interestingly, VOR gain measured with the vHIT seems not to be affected in Parkinson's disease (31). Parkinson's disease affects VOR function through multiple converging mechanisms: dopaminergic deficiency in brainstem and cerebellar circuits involved in VOR processing, impaired saccadic initiation and velocity, reduced cervico-ocular reflex contribution, and deficient multisensory integration. fHIT, by assessing the functional outcome of all these overlapping deficits simultaneously, provides a clinically relevant measure that may correlate better with balance function and fall risk than isolated kinematic measures. Its objective, quantitative nature makes it suitable for monitoring disease progression and for assessing response to dopaminergic therapy over time.

### 3.6 Vestibular prosthetics and intervention monitoring

The functional endpoint of fHIT, whether the patient can actually see the target during the head impulse, makes it a natural primary outcome measure for interventions aimed at restoring gaze stability rather than merely improving kinematic gain. The Maastricht group demonstrated this in a patient with bilateral vestibulopathy receiving a prototype vestibular implant delivering motion-modulated electrical stimulation to the lateral ampullary nerve. With the implant off, %CA ranged from 19–44%; with the implant on and motion-modulated stimulation active, %CA increased to 75–94% (*p* < 0.001) (32). This represented the first direct evidence that a vestibular prosthesis could restore functional dynamic visual acuity during high-acceleration head movements, not merely improve kinematic gain. The magnitude and gradient of the improvement also demonstrated fHIT's sensitivity to graded changes in vestibular afferent input, supporting its use for optimizing stimulation parameters in ongoing multi-patient implant trials and for comparing prototype performance across different stimulation protocols.

### 3.7 Athletic populations and normative data

Romano et al. (33) established sport-specific fHIT normative values in 124 professional athletes across six disciplines (American football, handball, ice hockey, soccer, bobsled/skeleton, and snowboarding). Handball players showed significantly higher %CA than the group as a whole (*p* < 0.001), while bobsled, skeleton, and snowboard athletes showed significantly lower vertical %CA (*p* < 0.001). Overall %CA declined with increasing acceleration, particularly evident above 6,001°/s^2^ horizontally and above 5,001°/s^2^ vertically, accelerations beyond the commonly tested range. Ball-sport athletes (American football, handball, ice hockey, soccer) showed higher vertical-plane %CA than non-ball-sport athletes across all acceleration bins, consistent with both selection for superior visuomotor integration and training-induced VOR adaptation to sports requiring rapid visual processing during high-acceleration play. The paradoxically lower vertical %CA in bobsled and snowboard athletes may reflect adapted central suppression of vestibular signals during sport-specific maneuvers involving extreme head accelerations, though this interpretation requires direct investigation (33).

Since sport-specific fHIT normative values are significantly different from those of the normal population, vestibular concussion in athletes should be evaluated on a sport specific normative range especially for return-to-play decisions. Cengiz et al. (18) provided comprehensive normative values for all six semicircular canals in 100 healthy young adults (aged 20–25 years) across the full acceleration range (1,000–7,000°/s^2^), confirming that 4,000–6,000°/s^2^ is the diagnostically optimal range, with mean %CA of 88–91% in healthy young adults. The normative data confirmed no significant side asymmetry in healthy individuals (establishing symmetry as a diagnostic expectation), modest sex-related differences emerging only above 6,000°/s^2^ (likely reflecting biomechanical rather than vestibular factors), and slightly higher vertical than horizontal %CA. The excellent test-retest reliability recently reported supports use of individual baseline values for longitudinal monitoring, making fHIT particularly suited to pre-season vestibular baseline testing and serial post-concussion follow-up (18).

## 4 Discussion

The cumulative evidence reviewed here suggests that the functional head impulse test occupies a fundamentally different position in vestibular assessment from that of its kinematic counterpart, the vHIT. While vHIT measures the *availability* of the vestibulo-ocular reflex drive, how well eye velocity tracks head velocity in the first 100 ms, fHIT measures something more consequential clinically: the *usability* of that drive within a real multisensory integration context. This distinction, initially implicit in the test's engineering rationale, has become progressively clearer as evidence has accumulated across populations and disorders, and it is arguably the central interpretive insight that the fHIT literature has produced.

The dissociation between vHIT gain and fHIT performance is not an incidental finding but a recurring structural feature of the data. It appears in acute vestibular neuritis, where fHIT asymmetry indices consistently exceed those of vHIT and correlate better with oscillopsia burden (7); in older people, where vHIT gain shows no significant difference between fallers and non-fallers while fHIT performance does, and correlates with the Tinetti Balance Score (27); in vestibular migraine, where VOR gain is typically preserved yet fHIT performance collapses under optokinetic conflict; and in dual-task paradigms, where adding concurrent cognitive load degrades fHIT percentage of correct answers while leaving vHIT gain statistically unchanged (20). Taken together, these converging dissociations point to the same interpretation: the clinical bottleneck in most real-world vestibular impairment is not the peripheral reflex arc itself, but the central capacity to integrate vestibular signals with visual and cognitive demands in real time.

**Figure 3:**
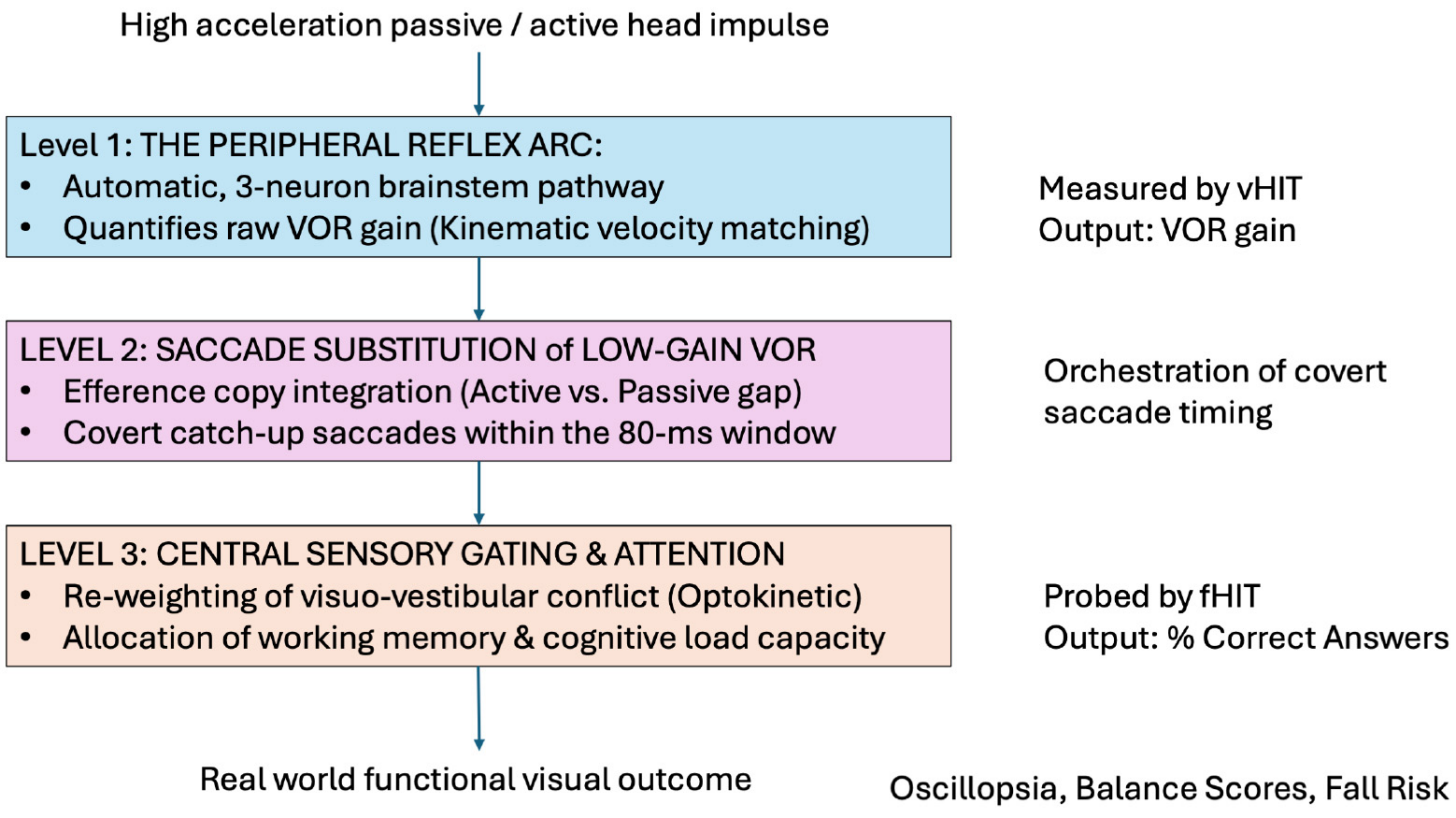
Schematic framework of the three functional levels governing gaze stabilization and real-world visual outcomes during head impulses. The diagram illustrates how high-acceleration head movements are sequentially processed across distinct central mechanisms, differentiating what is isolated by kinematic testing versus functional assessment. Level 1 (The Peripheral Reflex Arc): Represents the automatic, low-latency, three-neuron brainstem pathway. This level is quantified by the video head impulse test (vHIT), mapping raw vestibulo-ocular reflex (VOR) gain via kinematic velocity matching. Level 2 (Saccade Substitution of Low-Gain VOR): Explores the precise orchestration and timing of covert catch-up saccades. This level integrates central motor pre-programming and efference copy signals to trigger early, symbiotic corrective saccades—primarily within a critical 80-ms functional window—to substitute for an under-compensatory peripheral reflex during both active and passive head movements. Level 3 (Central Sensory Gating & Attention): Involves higher-order multimodal integration, requiring the real-time re-weighting of visuo-vestibular cues (e.g., during optokinetic conflict) and the allocation of cognitive and working memory resources under divided attention. While vHIT captures isolated peripheral reflex availability at Level 1, the functional head impulse test (fHIT) preferentially probes the entire pipeline through Level 3, yielding a behavioral metric (% correct answers) that correlates directly with real-world clinical impairments such as oscillopsia burden, balance scores, and fall risk.

**Figure 4:**
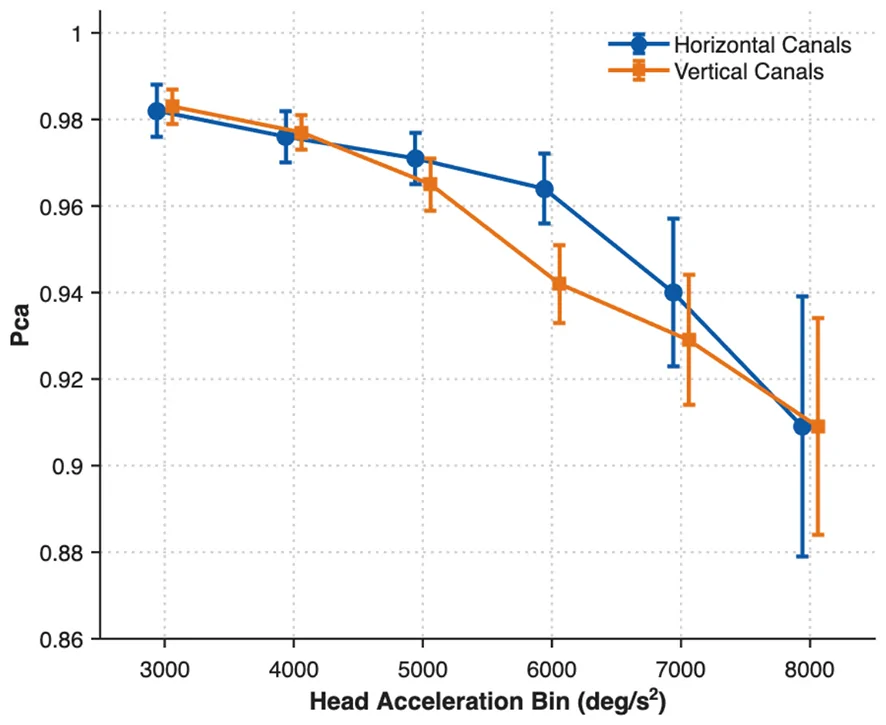
The diagnostic fHIT “vestibulogram” [redrawn from (33)]: Characterizing functional VOR thresholds across an ascending acceleration spectrum (1,000–7,000°/s^2^). As originally conceptualized by Romano et al. (33), this representation functions as a vestibular analog to the traditional audiogram, mapping a patient's visual stabilization performance capacity against progressively challenging physiological demands. In healthy cohorts, the envelope shows near-perfect percentage of correct answers (%CA) that remain stable up to extreme high-acceleration thresholds. Conversely, in pathological states, the curve drops precipitously. This functional boundary separates mechanical reflex availability (captured at low-to-mid accelerations) from the central integrative failure that triggers real-world oscillopsia and tracking errors when central sensory gating mechanisms break down. Error bars represent the standard deviation in %CA within each acceleration bin.

This interpretation can be organized into three functional levels (Figure 3). The first is the VOR arc proper, the canal afferent to brainstem to extraocular motor pathway that generates compensatory eye velocity. This level is what vHIT quantifies, and it is robust, automatic, and largely insensitive to attentional load. Much like an audiogram details a patient's sensory threshold across discrete acoustic frequencies, the acceleration-binned output of the fHIT functions as a functional vestibulogram. By plotting the percentage of correct answers against progressively demanding acceleration steps from 1,000 to 7,000°/s^2^ this vestibulogram charts the exact velocity-acceleration envelope where a patient's gaze-stabilization mechanics begin to fail under real-world processing demands (Figure 4). The second level involves the precise orchestration of covert saccade timing. While central motor pre-programming utilizes efference copy signals to command ultra-short-latency covert saccades during self-generated head movements, predictive higher-level control loops can trigger similar symbiotic corrective saccades even during passive, unpredictable movements (34–36). As demonstrated by the temporal constraints of the fHIT, the clinical utility of these saccades depends entirely on a tight timing window. If a saccade reduces gaze position error but lands after the 80-ms offset, it provides no functional visual benefit; however, when properly timed to cluster within the perceptual window—an ability preserved in some participants even under passive testing conditions—these saccades effectively rescue target recognition despite a severely reduced peripheral VOR drive. The third and most clinically decisive level is multimodal integration: the real-time reconciliation of vestibular, visual, and proprioceptive signals under conditions of competing sensory input and divided attention. It is this level that fHIT preferentially probes, because the task, recognizing a briefly flashed optotype during a passive, unpredictable head impulse, requires not only adequate gaze stabilization but also visual discrimination, attentional allocation, and short-term memory encoding, all within an 80 ms perceptual window. The sensitivity of fHIT performance to multimodal integration of vestibular, visual, and proprioceptive signals under conditions of competing sensory input and divided attention, such as optokinetic conflict in vestibular migraine, dual cognitive tasks in healthy subjects, and to the multifactorial complexity of postural control in older people with recurrent falls, reflects the engagement of this third level.

The clinical implications of this framework extend beyond diagnostic labeling. If what fHIT measures is the integrity of multisensory integration rather than peripheral VOR magnitude, then abnormal fHIT performance in the presence of normal vHIT gain, as seen in vestibular migraine, cognitively impaired older people, children with neurodevelopmental disorders, and Parkinson's disease, is not a paradox but a direct signal about the central processing layer. Similarly, the superior fall-risk prediction of fHIT compared to vHIT in the older people does not simply reflect greater test sensitivity; it reflects the fact that falls during daily life are themselves multisensory integration failures, demanding exactly the neural resources that fHIT taxes: multimodal integration of vestibular, visual and proprioceptive input; attentional demands during dual task; short-term memory, cognitive resources allocation. A test that mimics the integration demands of the real world will naturally predict real-world outcomes better than one that measures only the peripheral building block.

This also reframes the role of the compensatory saccades identified in vHIT. The critical variable is not their presence or their kinematic amplitude, but their *timing*: whether they arrive within the perceptual window of the fHIT stimulus. A covert saccade that reduces gaze position error but arrives after optotype offset contributes nothing to functional vision despite appearing effective on kinematic traces, while a saccade that arrives precisely during the 80 ms window can rescue recognition even in the context of severely reduced VOR gain. The fHIT outcome measure, percentage of correct answers, integrates all of these variables, reflex gain, saccade timing, saccadic suppression, visual processing speed, and attentional state, into a single functional verdict, which is why it tracks clinical reality more closely than any of its components measured in isolation. The clinical validity of this framework is further strengthened by independent replication from external centers; most notably, Casani and colleagues demonstrated that utilizing the fHIT alongside an optokinetic challenge successfully unmasks persistent, subclinical gaze-stabilization deficits in acute unilateral vestibulopathy participants even after structural symptom resolution, highlighting the test's unique capacity to probe central visuo-vestibular reliance beyond traditional kinematic metrics (21).

Looking forward, the fHIT framework raises a productive question for vestibular rehabilitation: if the test measures multisensory integration capacity rather than VOR gain alone, does rehabilitation that improves fHIT performance work by restoring peripheral VOR, by optimizing saccade timing, by improving central re-weighting of sensory conflict, or by enhancing attentional resources for gaze-stabilization tasks? The dual-task and optokinetic-conflict fHIT paradigms offer experimental tools to disentangle these contributions longitudinally. The field is now well-positioned to move from using fHIT as a diagnostic snapshot to using it as a window into the mechanisms of vestibular compensation and their cognitive dependencies.

## 5 Conclusions

The fHIT is a functional test that has a strong validation (versus the vHIT), normative data and a solid international literature regarding its utility in the measurement of the vestibular system's functional output. It has been adopted in assessing many vestibular and neurological disorders and correlates with quality of life. Despite the difficulties in defining all the complex neurophysiological processes that play a role in the fHIT paradigm, it has the advantage of having most of them measurable: VOR, visual acuity, attention, short-term-memory, cognitive deficit etc. leading the way to highlight the role of multisensory integration capacity. The fHIT could directly probe the higher-level central integrative loop by simultaneously challenging gaze stabilization, visual discrimination, attentional allocation, and short-term memory encoding during unpredictable passive perturbations. This theoretical shift reframes abnormal functional performance alongside normal kinematic gain not as a paradox, but as an indicator of central processing or cognitive resource allocation/attention deficits, providing a new blueprint for tailoring advanced vestibular rehabilitation.

## 6 Limitations

The major limitations of the fHIT are the functional nature of the test itself (it is a subjective not an objective test as vHIT) and the complex neurophysiological substrate that it explores. Since it is a subjective test, people can malinger at the test by simply choosing the wrong letter (as it also happens in pure tone audiometry with subjects reporting they cannot hear the sound). When analyzing the outcome of the fHIT is impossible to define the pathological weight of different neurophysiological processes such as multimodal integration of vestibular, visual and proprioceptive input; attentional demands during dual task; short-term memory and cognitive resources allocation. For this reason, it is fundamental to analyze the fHIT results based on the clinical diagnosis, the vHIT outcomes, and other functional measurements such as questionnaires.

## Data Availability

Requests to access the raw data supporting the conclusions of this article should be directed to Stefano Ramat, stefano.ramat@unipv.it.
